# Dexmedetomidine as adjuvant of transversus abdominal plane block for cesarean delivery under multimodal analgesia: a randomized clinical trial

**DOI:** 10.1186/s12871-026-03642-0

**Published:** 2026-01-23

**Authors:** Yiting Wang, Lini Wang, Yonghui Wang, Xiaoxiao Mu, Bingqing Zhao, Tingting Liu, Huang Nie

**Affiliations:** 1 Department of Anesthesiology and Perioperative Medicine, Xijing Hospital, Fourth Military Medical University, Xi’an, Shaanxi Province China; 2 Key Laboratory of Anesthesiology (The Fourth Military Medical University), Ministry of Education, Xi’an, China

**Keywords:** Cesarean delivery, Dexmedetomidine, Ropivacaine, Transversus abdominis plane block (TAPB), Multimodal analgesia

## Abstract

**Background:**

The parturients who have undergone cesarean delivery (CD) often suffer moderate to severe pain. Multimodal analgesia has been implemented to improve postoperative pain management following CD. The role of dexmedetomidine for multimodal analgesia after CD is not well defined. This study aimed to evaluate the efficacy of dexmedetomidine combined with ropivacaine in ultrasound-guided posterior transversus abdominis plane block(TAPB)on postoperative analgesia following CD.

**Methods:**

A total of 90 parturients scheduled for CD were divided randomly into two groups: The ropivacaine group (Group R), in which parturients were administered bilateral 0.25% ropivacaine 20 ml in posterior TAPB, and the dexmedetomidine group (Group RD), in which parturients were administered bilateral 0.25% ropivacaine and 0.5 µg/kg dexmedetomidine in posterior TAPB. Oral acetaminophen and celecoxib were routinely administered during the postoperative 48 h according to the multimodal analgesia protocol. The primary outcome was opioid consumption during the first 48 postoperative hours, and the secondary outcomes included opioid consumption during the postoperative 0-4,4–12,12–24,24–48 h, pain scores at 4,24,48 h, and time to first opioid administration.

**Results:**

Opioid consumption (morphine equivalents, MEQ) in the postoperative 48 h showed no significant difference in Group R and Group RD (median, 12.54 mg MEQ vs. 13.86 mg MEQ), *p* = 0.853. The 4 hours’ postoperative opioid consumption (MEQ) (median) was significantly lower in Group RD (median, 0 mg) for Group R (median, 1.32 mg, *p* = 0.012). Time to first opioid request was significantly prolonged in Group RD, *p* = 0.002. There was no significant difference in Numerical rating scale (NRS) score at rest and movement between the two groups. But the NRS score at uterine contraction was significantly lower in Group RD than Group R, *p* = 0.008.

**Conclusion:**

Dexmedetomidine as adjuvant of posterior bilateral TAPB in parturients under multimodal analgesia did not decrease the consumption of opioids, but prolonged time to first opioid request and decreased the NRS score at uterine contraction.

**Trial registration number:**

NCT05700045(the registration date: December 26,2022).

## Introduction

 Women undergoing cesarean delivery (CD) frequently experience moderate to severe postoperative pain. Previous studies have reported that the incidence of moderate to severe pain within the first 24 h after CD ranges from 40% to 90% [1, 2]. Inadequate pain management can delay maternal recovery, impair mother-infant bonding, and increase the risk of chronic pain or postpartum depression [3]. Traditional postoperative analgesia primarily relies on intravenous opioids, which are associated with adverse effects such as nausea, vomiting, pruritus, urinary retention, reduced gastrointestinal motility, and potentially life-threatening respiratory depression. Additionally, opioid use may pose risks to the newborn through breast milk transmission.

In light of these concerns, both the European Society for Regional Anesthesia and Pain Management and the Association of Obstetric Anesthesiologists recommend a multimodal analgesic approach for perioperative pain management in CD. This strategy, consistent with PROSPECT 2021 recommendations, is intended to optimize pain relief while minimizing opioid consumption [4]. Recommendations include the routine administration of acetaminophen and non-steroidal anti-inflammatory drugs (NSAIDs) during and after surgery, supplemented by regional anesthetic techniques such as the TAPB, with opioids reserved for rescue analgesia.

Ultrasound-guided TAPB delivers local anaesthetic to the neurovascular plane between the internal oblique and transversus abdominis muscles, interrupting afferent fibres from the T_6_–L_1_ spinal segments that supply the anterior–lateral abdominal wall. Consequently, TAPB provides effective analgesia for lower-abdominal surgery, including CD [5, 6]. Randomised data indicate that, in parturients who have not received intrathecal morphine, TAPB confers superior post-operative pain control compared with systemic analgesia alone. When intrathecal morphine is used, however, TAPB offers no incremental analgesic benefit. Nevertheless, women receiving TAPB alone regain bowel function and mobilise earlier, suggesting that avoidance of neuraxial opioids may accelerate recovery after CD. Two limitations restrict the clinical utility of single-shot TAPB: [1] inconsistent cranio-caudal spread produces incomplete sensory block in up to 30% of patients, and [2] the analgesic duration rarely exceeds 8–12 h.

Dexmedetomidine, a highly selective α_2_-adrenoceptor agonist, prolongs peripheral nerve block duration in a dose-dependent manner without increasing neurotoxicity [7, 8]. Whether dexmedetomidine added to ropivacaine for TAPB improves analgesia or reduces opioid requirement within an evidence-based multimodal regimen (regular paracetamol plus NSAIDs, rescue opioid) has not been tested in parturients. Previous work has largely examined TAPB in isolation, thereby might over-estimating treatment effects and failing to reflect contemporary practice [8–11]. Small, heterogeneous trials have yielded conflicting results, leaving the value of dexmedetomidine-enhanced TAPB after CD uncertain.

Previous meta-analyses have indicated that the posterior TAPB provides more prolonged analgesia compared to the lateral approach [12]. Furthermore, local anesthetic administered via the posterior approach can diffuse into the paravertebral region, potentially alleviating visceral pain. Therefore, we conducted a randomised, blinded, controlled trial to determine whether the addition of dexmedetomidine to ropivacaine in an ultrasound-guided posterior TAPB would reduce cumulative 48-hour opioid consumption and improve analgesic outcomes—including pain scores during uterine contractions—when superimposed on a standard multimodal analgesic protocol (regular paracetamol and NSAIDs with rescue opioids) following cesarean delivery (CD).

## Methods

Ethical approval was obtained from the regional ethics committee of the Fourth Military Medical University Xijing Hospital (XJLL-KY-20222263-F-1). The study was registered at Clinical Trials. gov (NCT05700045, December 26, 2022), the complete plan and analysis can be obtained through the public channels of the platform. The study was conducted according to the Consolidated Standards of Reporting Trials statement. This single-center, randomized, double-blind, parallel-controlled clinical study was conducted at the Fourth Military Medical University Xijing Hospital, from May 2023 to September 2023. The first participant was enrolled in the trial on May 31, 2023. Written informed consent was obtained from all participants before enrolling for the study.

### Study design

Eligible participants were those Age > = 18 years, ASA (American Society of Anesthesiologists) grade I-III, 37–42 weeks of gestation, scheduled for CD and postoperative PCIA analgesia, participate in the trial voluntarily and obtain informed consent. Exclusion criteria were CD with general anesthesia or neuraxial anesthesia with epidural administration, the combination of other opioids during operation, high-risk pregnancy (multiple pregnancies, in vitro fertilization, etc.) or pregnancy-related complications (hypertension, preeclampsia, chorioamnionitis, etc.), the times of previous CD ≥ 3, BMI ≥ 50 kg/m^2^, hypersensitivity or contraindications to the drugs involved in the study, combined with surgery other than tubal ligation and oophorectomy, severe renal impairment (SCr > 176 µmol/L and/or eGFR < 60 L/min), severe liver impairment (ALT and/or AST more than 3 times the upper limit of normal value), coagulation dysfunction or increased bleeding risk (PLT < 80 × 10^9^/L or INR > 1.5), history of chronic pain or opioid abuse, enrolled in other clinical trials in the past three months. Trained research staff identified and screened all potential participants during their pre-anaesthetic evaluation.

On arrival in the operating room, a 16-G peripheral venous cannula was inserted in the upper limb and routine monitoring (non-invasive blood pressure, 3-lead ECG, pulse oximetry) was instituted; oxygen 3 L min⁻¹ was delivered via a mask. Spinal anaesthesia was performed in the lateral position with hyperbaric bupivacaine 0.75% 1.4–1.6 mL plus 50% glucose 0.2–0.3 mL, the exact volume being left to the discretion of the blinded anaesthetist after consideration of maternal height. An epidural catheter was inserted only if surgery lasted > 90 min. Immediately after cord clamping, all participants received: Dexamethasone 10 mg i.v, Parecoxib 40 mg i.v. and Palonosetron 0.25 mg i.v.

At the end of surgery, participants were transferred to the post-anaesthesia care unit (PACU) where the study TAPB was performed. On arrival in the ward, a wireless PCIA pump (apon ZZB-IV-100 with iPainfree^®^ cloud monitoring) was connected. The pump contained hydromorphone 10 mg diluted to 100 mL with 0.9% saline, settings were: no background infusion, bolus 2 mL, lock-out 10 min.

Standard multimodal analgesia comprised: Paracetamol 300 mg orally TDS for 48 h.

and Celecoxib 200 mg orally BD for 48 h. Rescue analgesia was provided by the anaesthetist if: NRS at rest > 3, or NRS on movement > 6. Rescue medication was i.v. hydromorphone 0.3–0.5 mg. All hydromorphone consumed (PCIA + rescue) was recorded and converted to intravenous morphine equivalents (MEQ) using a 5:1 ratio.

### Blinding and randomization

Randomization was performed at the end of surgery to ensure that no excluded medications had been administered. A computer-generated randomisation list (R v4.2.1; 1:1 allocation; variable block sizes of 4 and 6) was prepared by an independent statistician and transferred to sequentially numbered, opaque, sealed envelopes.

A nurse who had not been involved in intra-operative care confirmed the integrity of the envelope and opened it, prepared the study drug in 50 mL syringes of identical appearance labelled only “study injection”. Participants were thereby allocated to: Group RD: posterior TAPB with 20 mL 0.25% ropivacaine + dexmedetomidine 0.5 µg kg⁻¹ per side; Group R: posterior TAPB with 20 mL 0.25% ropivacaine per side. The syringes were handed to an anaesthetist who performed the blocks. Blinding was assessed by querying and observing subjects and study personnel regarding their ability to identify treatment groups and their associated behavioral responses. Investigators, participants, ward staff, outcome assessors and the statistician were blinded to treatment allocation throughout. Data were analysed as “A” versus “B”; the randomisation code was broken only after the database was locked and the primary analysis completed.

### TAPB procedure

Posterior TAPB was performed in the PACU by a single fellowship-trained regional anaesthetist with > 5 years’ experience. Standard monitoring (3-lead ECG, pulse oximetry, non-invasive blood pressure) was maintained throughout. With the parturient in the semi-lateral position, the skin was disinfected with 2% chlorhexidine in 70% isopropyl alcohol. A high-frequency linear probe (12 MHz) was placed perpendicular to the skin at the mid-axillary line and the abdominal wall layers were identified. The probe was moved posteriorly until the Petits triangle (bordered by the iliac crest, latissimus dorsi and external oblique) and the quadratus lumborum muscle came into view. Using an in-plane technique, a 22-G, 100-mm block needle was advanced until the tip lay between the posterior aspect of the transversus abdominis muscle and the quadratus lumborum [12], 2 mL 0.9% saline was injected to confirm correct separation of these fascial planes. The study solution was then injected slowly in 5 mL aliquots, with gentle aspiration between each, while real-time ultrasound confirmed cranio-caudal spread. A still image and a 5-s cine-loop were stored for later review.

Block quality was graded independently by a second senior regional anaesthetist who was blinded to group allocation:

- Good: clear hypoechoic oval spread separating transversus abdominis and quadratus lumborum
- Average: partial separation or streaky spread
- Poor: no identifiable fluid collection

### Outcome measures

Primary outcome was cumulative post-operative opioid consumption in the first 48 h, expressed as intravenous morphine equivalents (MEQ) and extracted automatically from the PCIA pump. Time-zero (T₀) was defined as the moment the study TAPB injection was completed. Secondary analgesic outcomes included : NRS pain scores (0–10) at 4, 24 and 48 h after T₀, recorded: at rest; on movement (90° roll); during uterine contraction (highest score evoked by breastfeeding or exogenous oxytocin in the interval); cumulative MEQ in the intervals 0–4, 4–12, 12–24 and 24–48 h; time to first post-T₀ opioid request (minutes).

Recovery and safety variables were time to first ambulation (minutes from T₀); opioid-related adverse events within 48 h such as hypotension (SBP < 90 mmHg or DBP < 50 mmHg or decrease beyond 20% of baseline), bradycardia (HR < 60 bpm), respiratory depression (RR < 10 min⁻¹ or SpO₂ < 90%), pruritus, post-operative nausea/vomiting (PONV), dizziness. Sedation level at 4, 24 and 48 h using the Ramsay Sedation Scale (RSS 1–6, RSS>3 sedation). Maternal satisfaction at 48 h (0 = unsatisfactory, 1 = satisfactory, 2 = highly satisfactory).

### Statistical analysis and sample size calculation

Sample size estimation was based on the data in previous similar studies [5], the consumption of hydromorphone at 48 h after the operation was normally distributed, and the sample size can be calculated by using the difference test, and the consumption of opioids at 48 h after operation in the control group ( Expressed in morphine equivalent), the mean value is 20.8 mg, the standard deviation is 4.18, and the intervention group is expected to reduce by 15% [13], it is calculated by PASS 2015 software that at least 80 patients need to carry out the difference test to have 90% power to identify significant differences at the level of α = 0.05 (two-sided test). Considering the dropout rate of 10%, at least 90 patients, 45 in each group, should be included in this study. Normality was tested using the Shapiro-Wilk (SW) test, ordinal data and continuous data were analyzed using Student’s t-test and Wilcoxon tests depending on the distribution type. For categorical data, we applied the χ2 test or Fisher’s exact test as appropriate. Variables were presented as mean (SD), median (IQR) count (%). The Mann-Whitney U test, a nonparametric method for comparing two independent groups, was used to compare the postoperative hydromorphone consumption (MEQ) and the cumulative number of bolus attempts between the groups. Besides the Log-Rank test was performed at the time of first opioid administration, and the cumulative events curve of the time to first opioid administration was plotted. A generalized linear mixed model (GLMM) was used to compare the resting, activity and uterine contraction pain NRS scores between the two groups. Subgroup analyses were performed for the primary outcome based on three baseline variables (gestational diabetes mellitus, previous CD, and BMI> 28 kg/m^2^). The quality of TAPB, whether oral analgesics were taken on time and remedial analgesia after CD were used as the sensitivity analysis for the primary outcome. All of the following statistical analyses were performed using SPSS 26.0 statistical software, the level of statistical significance was 5% and all hypothesis tests were performed with two-sided tests.

## Results

**Fig. 1:**
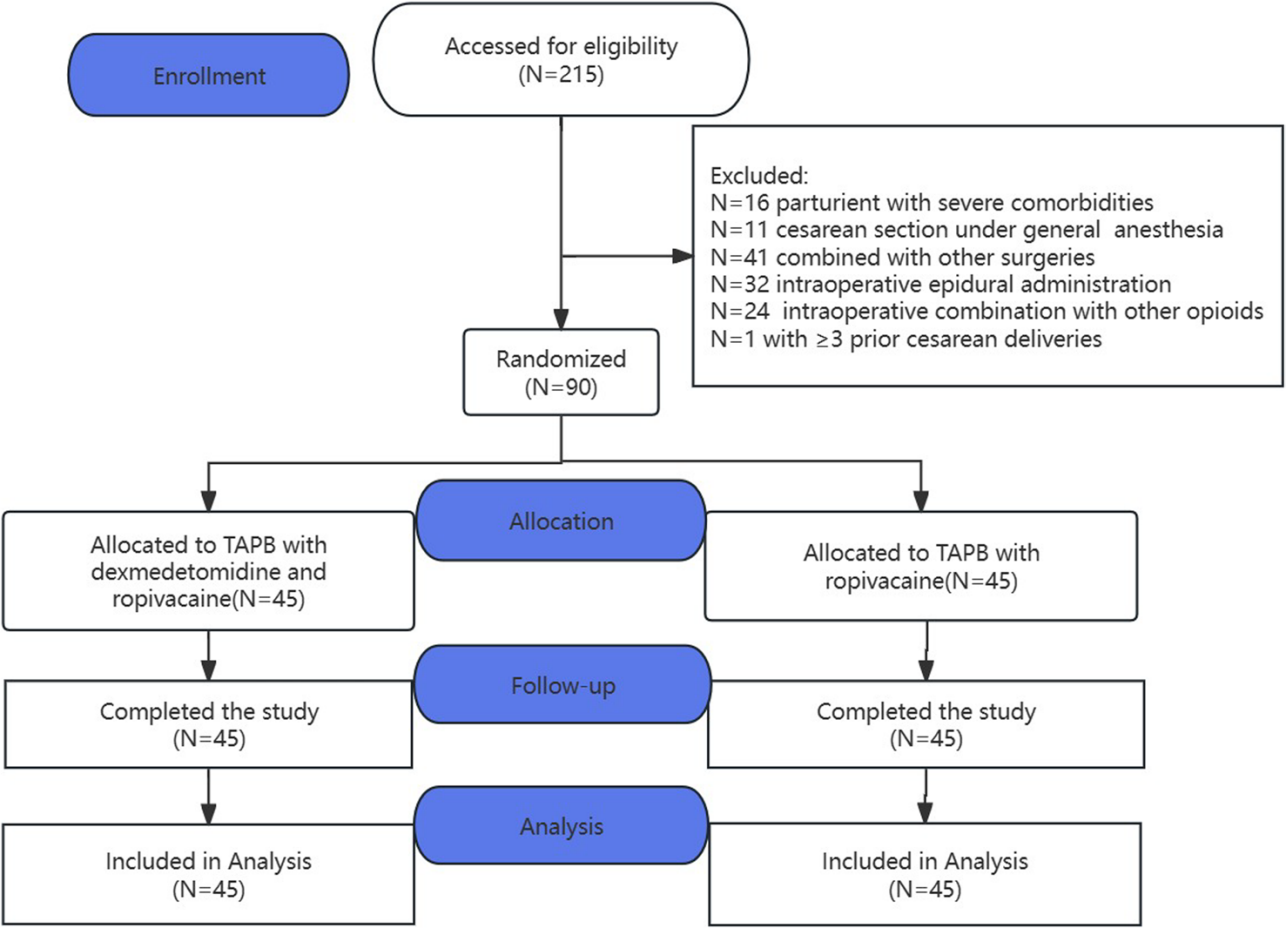
Consolidated Standards of Reporting Trials (CONSORT) flow diagram. TAPB, transmuscular abdominal block

215 parturients scheduled for CD at the First Affiliated Hospital of Air Force Military Medical University from May 2023 to September 2023 were screened for eligibility and 90 participants were enrolled. The flow of the study participants is shown in Fig. 1. There were no statistically significant differences in demographic and perioperative characteristics between the two groups (Table 1).

**Table 1:** Demographic and perioperative characteristics

Characteristics	Group RD (*N* = 45)	Group *R* (*N* = 45)	*P*
Age, yr mean (SD)	30.44(3.72)	31.40(4.21)	0.257
Gestational week, w mean (SD)	38.9(0.95)	38.76(1.11)	0.477
BMI, kg/m^2^, mean (SD)	28.06(3.58)	27.73(3.21)	0.649
Education degree, n (%)			0.407
High school and below	7(15.5)	3(6.6)	
College and above	38(84.5)	42(93.4)	
Parity, n (%)			0.610
1	33(73.3)	29(64.4)	
2	11(24.4)	14(31.1)	
3	1(2.2)	2(4.4)	
ASA, n (%)			>0.999
I	0(0.0)	0(0.0)	
II	45(100.0)	44(97.8)	
III	0(0.0)	1(2.2)	
Emergency, n (%)	21(46.7)	19(42.2)	0.671
Comorbidities, n (%)			0.267
no	21(46.7)	27(60.0)	
PROM	4(8.9)	1(2.2)	
PIH	0(0.0)	1(2.2)	
GDM	7(15.6)	7(15.6)	
Hypothyroidism	11(24.4)	5(11.1)	
others	2(4.4)	4(8.9)	
Hypoalbuminemia, n (%)	27(60.0)	35(77.8)	0.069
Combined operation, n (%)			>0.999
no	42(93.3)	41(91.1)	
Uterine artery Ligation	3(6.7)	2(4.4)	
Lysis of pelvic adhesions	0(0.0)	1(2.2)	
others	0(0.0)	1(2.2)	
Incision, n (%)			0.078
transverse incision	42(93.3)	45(100.0)	
vertical incision	3(6.7)	0(0.0)	
Duration of surgery, min mean (SD)	69.71(12.89)	67.84(14.10)	0.514
Puncture intervertebral space, n (%)			0.581
L2-3	9(20.0)	7(15.6)	
L3-4	36(80.0)	38(84.4)	
Hypotension during the operation, n (%)	13(28.9)	18(40.0)	0.267
Vasoactive drugs, n (%)	42(93.3)	43(95.6)	0.645
Crystalloid, ml, median (IQR)	1100(1100, 1600)	1100(1100, 1600)	0.529
Blood loss, ml, median (IQR)	200(200, 200)	200(200, 200)	0.677
Urine output, ml, mean (SD)	143.11(81.76)	156.67(77.3)	0.421
TAPB quality, n (%)			0.433
poor	4(8.9)	1(2.2)	
fair	18(40)	21(46.7)	
good	23(51.1)	23(51.1)	

**Fig. 2:**
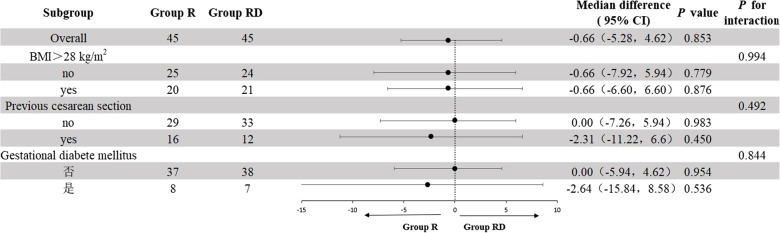
Primary outcome subgroup analysis

BMI, body mass index; ASA, American Society of Anesthesiologists;

PROM, pre-labor rupture of membranes; PIH: hypertension in pregnancy;

GDM, gestational diabetes mellitus;

There was no statistically significant difference in total opioid consumption during the first 48 postoperative hours between the two groups. Median (IQR) morphine-equivalent consumption was 13.86 (7.26–20.13) mg in Group RD versus 12.54 (6.27–24.42) mg in Group R (*p* = 0.853, Table 2). Three pre-planned sensitivity analyses yielded identical conclusions: (1) after exclusion of 5 participants with poor-quality TAPB; (2) after exclusion of 9 participants who omitted ≥ 1 scheduled dose of oral analgesic; 3)after exclusion of 8 participants who received rescue opioid. In all scenarios, median consumption remained comparable between groups (Table 2). Likewise, none of the pre-specified subgroup analyses (age, BMI, previous CD, intrathecal opioid use) showed a significant interaction with treatment allocation (Fig. 2).

**Table 2:** Primary and secondary outcomes

	Group RD	Group *R*	Median difference (95%CI)	*P*
Primary outcome				
MEQ 0–48 h, median (IQR)	13.86 (7.26–20.13)	12.54 (6.27–24.42)	-0.66 (-5.28,4.62)	0.853
Primary outcome sensitivity analysis				
TAPB quality^1^(*N* = 85)	13.86 (7.26–21.45)	12.54 (6.93–24.42)	-0.66 (-5.28,4.62)	0.864
Oral NSAIDs on time^2^(*N* = 81)	14.19 (7.59–20.95)	11.22 (4.62–25.74)	1.32 (-4.62,5.94)	0.732
Unremedial analgesia^3^(*N* = 82)	13.86 (7.42–19.80)	10.8 (4.95–22.70)	0.66 (-3.96,5.28)	0.759
Secondary outcomes				
MEQ 0–4 h, median (IQR)	0 (0-1.98)	1.32 (0-3.30)	-1.32 (-1.32,0)	0.012
MEQ 4–12 h, median (IQR)	1.32 (0-3.30)	1.32 (0-3.63)	0 (-0.66-0.66)	0.937
MEQ 12–24 h, median (IQR)	3.30 (1,32-5.94)	3.96 (1.32–7.59)	0 (-1.98,1.32)	0.535
MEQ 24–48 h, median (IQR)	7.26 (3.96–12.54)	5.94 (2.97–11.55)	0.66 (-1.98,2.64)	0.671
NRS at rest^4^				0.482
4 h postoperatively, median (IQR)	1(0–2)	2(0–2)	0(-1,0)	
24 h postoperatively, median (IQR)	0(0–2)	0(0–2)	0(0,0)	
48 h postoperatively, median (IQR)	0(0–0)	0(0–0)	0(0,0)	
NRS at movement^4^				0.225
4 h postoperatively, median (IQR)	3(2–4)	3(2–4)	0(-1,1)	
24 h postoperatively, median (IQR)	4(2.5–5.5)	4(3–5)	0(-1,0)	
48 h postoperatively, median (IQR)	3(2–4)	3(2–4)	0(-1,0)	
NRS at uterine contraction^4^				0.008
4 h postoperatively, median (IQR)	1(0–3)	3(1–5)	-1(-2,0)	
24 h postoperatively, median (IQR)	1(0–3)	3(1–5)	-1(-2,0)	
48 h postoperatively, median (IQR)	0(0–1)	0(0–2)	0(0,0)	
Time to first PCA in hours, median (IQR)	4.45 (2.15–12.91)	1.52 (0.53–5.36)	2.28 (1.05,3.88)	0.002

CI, confidence interval MEQ, postoperative hydromorphone use in morphine equivalents; PCA, patient-controlled analgesia

1.TAPB quality: 5 parturients with poor TAPB were excluded (41 parturients in Group RD and 44 parturients in Group R) for sensitivity analysis

2. Oral NSAIDs on time: 9 parturients who did not take oral analgesics on time after surgery were excluded (42 parturients in Group RD and 39 parturients in Group R) for sensitivity analysis

3. Unremedial analgesia: 8 parturients with remedial analgesia after surgery were excluded (42 parturients in Group RD and 40 parturients in Group R) for sensitivity analysis

4. As the outcome was repeated measures and skewed, a generalized linear mixed model was used for comparison

Median (IQR) morphine-equivalent consumption during the first 4 h was significantly lower in Group RD: 0 (0–1.98) mg versus 1.32 (0–3.30) mg in Group R (*p* = 0.012, Table 2). No between-group differences emerged in any subsequent 12-h epoch (Table 2).

**Fig. 3:**
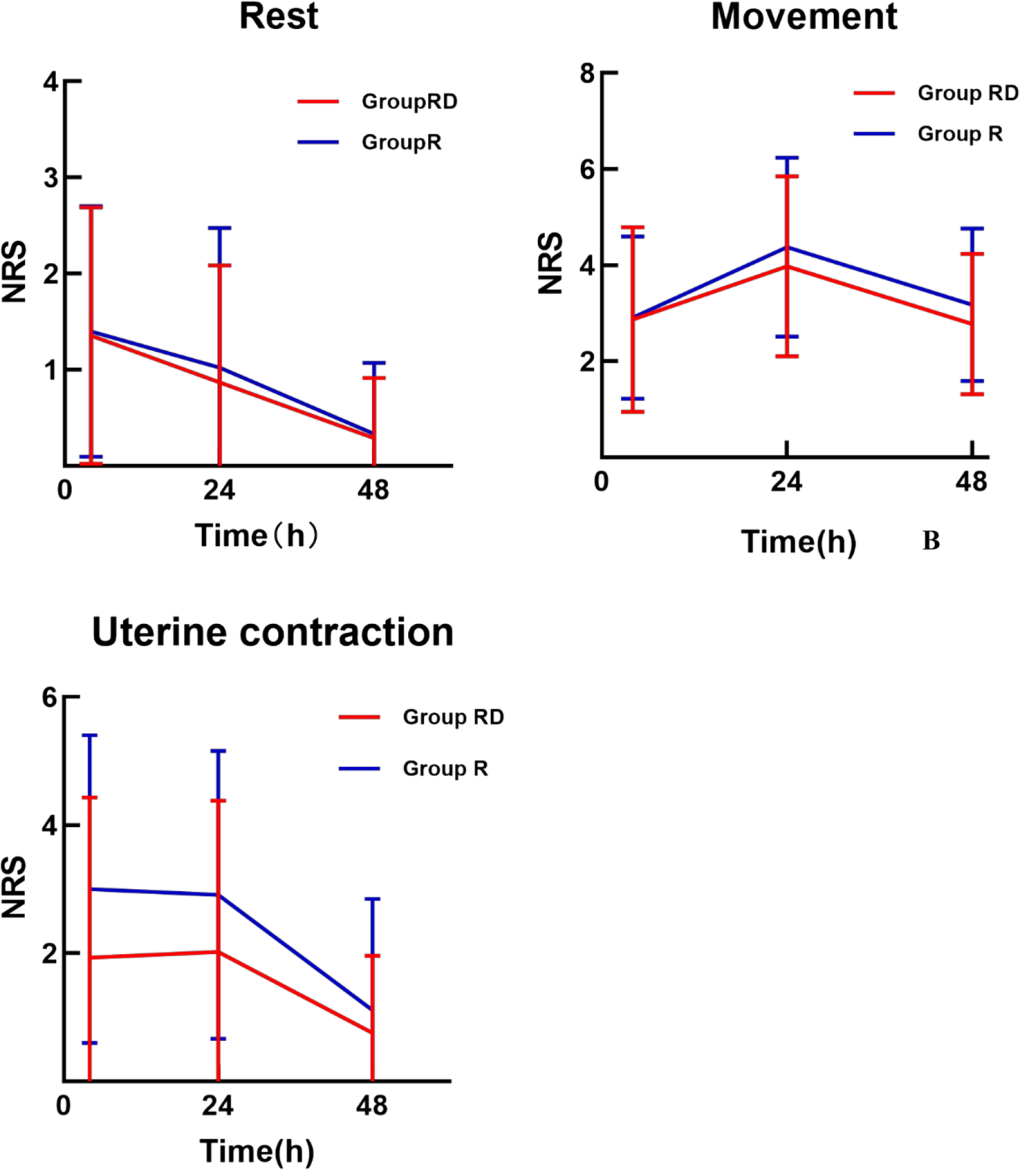
Pain score trajectories over time

NRS scores (heavily right-skewed and repeated-measures) were analysed with a generalised linear mixed model. The postoperative pain score trajectories over time was shown in Fig. 3. Rest and movement: no treatment effect (*p* ≥ 0.26). Uterine contraction: mean NRS consistently lower in Group RD (*p* = 0.008, Table 2).

**Fig. 4:**
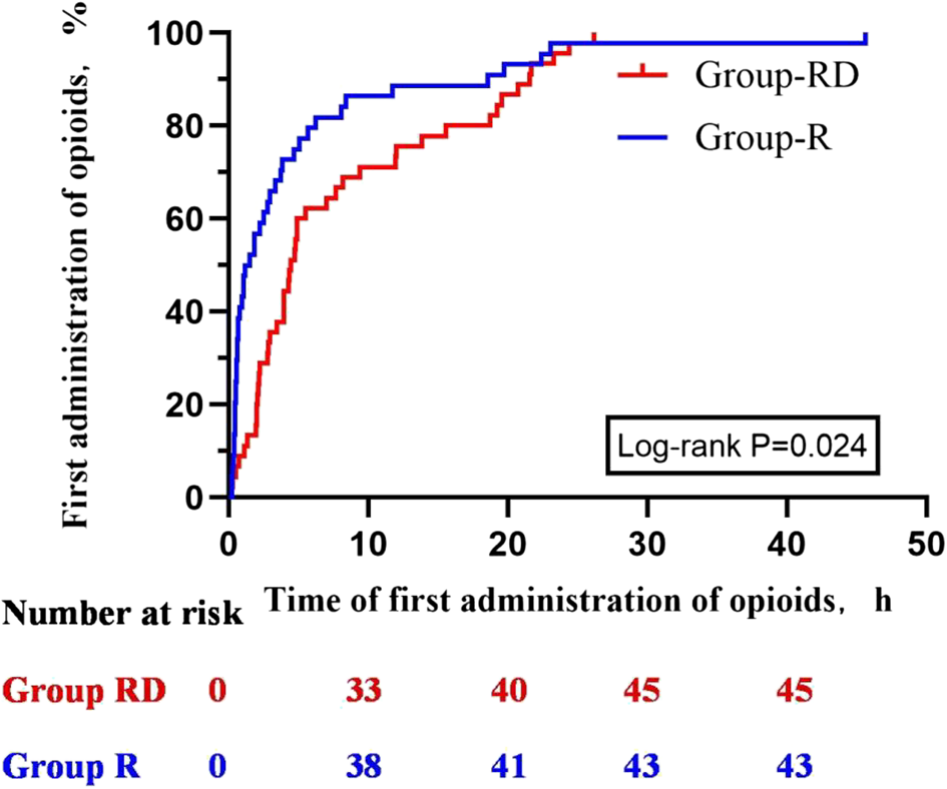
Cumulativie events plot of time to first opioid

Time to first opioid request was prolonged in Group RD: median 4.45 h (2.15–12.91) vs. 1.52 h (0.53–5.36) in Group R (Kruskal–Wallis *p* = 0.002; log-rank *p* = 0.024, Fig. 4).

There was no significant difference in postoperative characteristics such as incidence of opioid-related adverse events, time to first ambulation, rescue analgesic and parturient satisfaction between the two groups (Table 3).

**Table 3:** Postoperative recovery outcomes

	Group RD	Group R	P
Bradycardia, n (%)	7(15.6)	5(11.1)	0.535
Hypotension, n (%)	1(2.2)	2(4.4)	>0.999
Respiratory depression, n (%)	0	0	NA
Dizziness, n (%)	0	0	NA
Nausea, n (%)	0	0	NA
Vomit, n (%)	0	0	NA
Pruritus, n (%)	0	0	NA
sedation, n (%)	0	0	NA
Rescue analgesic, n (%)	3(6.7)	5(11.1)	0.714
Time for first ambulation in hours, mean (SD)	22.27(3.74)	22.24(3.99)	0.977
Parturient satisfaction			0.082
Unsatisfactory, n (%)	0(0.0)	0(0.0)	
Satisfactory, n (%)	13(28.9)	21(46.7)	
Highly Satisfactory, n (%)	32(71.1)	24(53.3)	

## Discussion

This randomized comparative trial demonstrated that the addition of dexmedetomidine to ropivacaine for TAPB did not reduce postoperative opioid consumption or decrease NRS scores at rest or during movement following CD, compared with ropivacaine alone. However, the combination did prolong the time to first opioid administration and reduced NRS scores during uterine contractions.

There was no statistically significant difference in total morphine equivalent (MEQ) consumption (median: 13.86 mg in Group RD vs. 12.54 mg in Group R) during the 48-hour postoperative period. Consequently, the analgesic effect was more modest than anticipated, failing to achieve the expected 15% reduction. Several factors may explain this outcome. First, the implementation of multimodal analgesic strategies during CD significantly attenuated postoperative nociception and opioid use in both groups, likely diminishing intergroup differences. As demonstrated in a comprehensive study by Nedeljkovic et al., which compared TAPB using 266 mg liposomal bupivacaine plus 50 mg bupivacaine HCl versus 50 mg bupivacaine HCl alone for post-cesarean analgesia under neuraxial anesthesia, the 48-hour MEQ consumption in both our intervention (13.86 mg) and control (12.54 mg) groups was notably lower than the 20.5 mg reported in their study [5]. Similarly, compared with another comparable study, our 24-hour oral morphine equivalent consumption (17.82 mg in Group RD vs. 21.78 mg in Group R) was markedly lower (65.3 mg in the referenced study) [14]. Compared with a meta-analysis published in 2025(41.67 mg in TAPB vs. 26.15 mg in ITM) [15], the 24 h OME (17.82 mg in Group RD vs. 21.78 mg in Group R) in the present study was significantly lower. These findings suggest that standard multimodal analgesia may have obscured potential differences between groups. Second, the relatively small sample size may have limited the statistical power to detect differences in primary and secondary outcomes, such as opioid consumption and NRS scores. Thus, larger-scale studies are warranted to further evaluate the efficacy of dexmedetomidine as an adjunct in TAPB for post-CD analgesia.

Dexmedetomidine is widely regarded as an ideal adjuvant to local anesthetics in various regional anesthesia techniques. Mohta et al. [16] reported that 1 µg/kg dexmedetomidine used as an adjunct to 0.5% bupivacaine in paravertebral nerve block significantly prolonged paravertebral nerve block duration, reduced opioid consumption, and decreased the incidence of postoperative nausea and vomiting. Similarly, Aksu et al. [17] also demonstrated that dexmedetomidine combined with bupivacaine in TAPB could decrease postoperative pain and opioid consumption after lower abdominal surgery. However, a newly published meta-analysis finding that adjuvants including dexmedotomedine [18] had minimal clinical impact on postoperative opioid consumption after cesarean section supported our result.

In the present study, the time to first opioid request was significantly shorter in Group R (median: 1.52 h) compared with Group RD (median: 4.45 h), aligning with the results of previous studies. The finding indicated that dexmedetomidine, when used as an adjuvant for posterior TAPB, extended the duration of local anesthetic action, enhanced analgesic efficacy, and reduced early postoperative opioid consumption. This duration is longer than that reported by Joseph and Abdelaal et al. [19]. (mean: 130 min), but shorter than the findings of Haitao Qian et al. [10]. (mean: 9.62 h). Prior studies have confirmed the efficacy of 0.25–0.5% ropivacaine for TAPB [20, 21]. In one such study, the intervention group received 0.5 µg/kg dexmedetomidine added to 0.3% ropivacaine, which had a higher concentration of local anesthetic than used in our study, potentially accounting for the longer pain-free duration observed. The duration of opioid request delay required to achieve clinical significance has not been rigorously established in the literature. According to a newly published meta-analysis [18] a delay of 3 h is considered the minimal clinically important difference. Our findings thus indicate a clinically significant prolongation. The mechanism by which dexmedetomidine prolongs opioid analgesic effect of TAPB is thought to involve both peripheral and central pathways. It has been hypothesized that dexmedetomidine may delay the systemic absorption of local anesthetics by activating peripheral α₂-adrenergic receptors, leading to vasoconstriction at the injection site and thereby extending the duration of neural blockade [22]. Additionally, other research has indicated that systemically absorbed dexmedetomidine may exert central analgesic effects by acting on the locus coeruleus, further contributing to its analgesic efficacy and potentially influencing hemodynamic stability [23].

Postpartum uterine contraction pain is a visceral pain which is often related to the use of postpartum oxytocin, postpartum breast-feeding and other factors. Uncontrolled uterine contraction pain affect maternal postoperative recovery and increase maternal risk of postoperative depression [24, 25]. Dexmedetomidine has demonstrated established efficacy in mitigating visceral nociception. Systemic infusion of this agent has been shown to abolish colorectal-distension pain in rat models, while clinical evidence indicates that it suppresses uterine contractility through inhibition of oxytocin release [26–28]. A dexmedetomidine–oxycodone combination further reduces visceral pain during major abdominal surgery [29]. In this trial, NRS scores during active uterine cramping were lower in the dexmedetomidine–ropivacaine (RD) group than in the ropivacaine-only (R) group. This suggests that perineural dexmedetomidine, when administered as an adjunct in TAPB, may confer visceral antinociceptive benefits and facilitate opioid-sparing analgesia. However, these results should be interpreted with caution and warrants dedicated investigation since NRS was not the primary outcome in this study.

Bradycardia and hypotension are the most common side effects of dexmedetomidine when administered intravenously [30]. The results demonstrated a slight increase in bradycardia in the two groups, which was not significantly different between the groups. Regarding postoperative hypotension, the hypotension of 1 parturient in the control group was associated with postpartum hemorrhage. Additionally, we did not observe more severe bradycardia (heart rate < 50 beats/min) in this study. Due to the low dosage of opioids after CD in this study, we also did not observe opioid-related adverse effects such as pruritus, postoperative nausea and vomiting, dizziness, and respiratory depression.

## Limitations

There were several limitations to our study. First, the randomized controlled double-blinded trial was conducted at a single center, which could potentially impinge upon the external validity of the findings. Further clinical trials are required at multiple centers to generalize the results. Second, our study did not determine whether dexmedetomidine acts primarily through peripheral α₂-receptors or via systemic uptake. Plasma concentration monitoring was not conducted; thus, further dose-finding studies remain necessary. Third, concomitant spinal anesthesia precluded formal sensory testing, leading us to rely on opioid consumption as the primary outcome. Finally, neither umbilical cord nor serial neonatal blood levels were measured, and routine observations—including heart rate, respiration, SpO₂, and neurobehavioral scores—were not recorded. Consequently, the potential fetal–neonatal effects remain uncharacterized. However, studies have shown that dexmedetomidine is beneficial for the induction and maintenance of general anesthesia in obstetrics and is safe for the fetus and newborn [31]. In this study, dexmedetomidine was only used as an adjuvant to local anesthetics, with blood drug concentrations far lower than those achieved through intravenous administration, thus theoretically it would not affect the safety of the mother or baby.

In conclusion, under multimodal analgesia background, adding 1 µg kg⁻¹ dexmedetomidine to 0.25% ropivacaine for TAPB after CD prolonged the time to first analgesic request and reduced uterine-contraction pain scores, but did not decrease 48 h opioid consumption or NRS scores at rest or on movement. Dexmedetomidine may be selectively considered as adjunct for TAPB in parturients with high risk of suffering uterine contraction pain. Larger, mechanistically oriented trials are warranted to define the place of dexmedetomidine in multimodal post-caesarean analgesia.

## Data Availability

All data generated or analysed during this study are included in this published article.

## Abbreviations

CD: cesarean delivery
NRS: numeric rating scale
NSAIDs: non-steroid anti-inflammatory drugs
PCIA: patient-controlled intravenous analgesia
TAPB: transversus abdominis plane block
